# Construction of a prognostic risk model based on apoptosis-related genes to assess tumor immune microenvironment and predict prognosis in hepatocellular carcinoma

**DOI:** 10.1186/s12876-022-02481-w

**Published:** 2022-08-26

**Authors:** Xiqin Wang, Chenguang Ji

**Affiliations:** 1 Department of Gastroenterology, The Second Hospital of Hebei Medical University, No. 215, Heping West Road, Shijiazhuang, 050000 Hebei China; 2 Internal Medicine, Yuhua Yunfang Integrated Traditional Chinese and Western Medicine Clinic, Shijiazhuang, China

**Keywords:** Hepatocellular carcinoma, Apoptosis, Prognosis, Signature, Nomogram

## Abstract

**Background:**

Hepatocellular carcinoma (HCC) is a serious malignant disease with high incidence, high mortality and poor prognosis. This study aimed to establish a novel signature based on apoptosis-related genes (ARGs) to predict the prognosis of HCC.

**Methods:**

Expression data of HCC from TCGA database and the list of 160 ARGs from MSigDB were downloaded. The genes included in apoptosis-related signature were selected by univariate Cox regression analysis and lasso Cox regression analysis. Subsequently, a prognostic risk model for scoring patients was developed, and then separates patients into two groups. Kaplan–Meier and receiver operating characteristic analysis were performed to evaluate the prognostic value of the model in TCGA, GEO and ICGC databases. The characteristics of immune cell infiltration between two groups of HCC were investigated. Finally, a nomogram was plotted to visualize the prognosis prediction.

**Results:**

Nine genes (CDC25B, DAP3, ETF1, GSR, LGALS3, MGMT, PPP2R5B, SQSTM1 and VDAC2) were included in the prognostic risk model. Survival was lower in the high-risk group. Surprisingly, the high-risk group was significantly more in immune cell infiltration and with higher immunoscore and stromalscore than in the low-risk group. In addition, the risk score was an independent prognostic factor for HCC.

**Conclusions:**

Prognostic signature comprising nine ARGs could be used as a potential prognostic factor for HCC. It also provides an important idea for further understanding the immunotherapy of HCC.

**Supplementary Information:**

The online version contains supplementary material available at 10.1186/s12876-022-02481-w.

## Introduction

Primary liver cancer is one of the six most common cancer and the third leading cause of cancer death [1]. In patients with primary liver cancer, hepatocellular carcinoma (HCC) accounts for about 75–85% [1]. Hepatitis B virus (HBV) and hepatitis C virus (HCV) are major risk factors for HCC [2]. Despite multiple management strategies are available for treatment HCC, the relapse rates of HCC remain high and the survival is poor [3]. In view of this, it is urgent to rely on reliable prognostic markers to build prognostic models to improve the accuracy of prognosis, which is conducive to the development of personalized treatment.

Apoptosis, also known as programmed cell death, is finely regulated at the gene level resulting in the orderly and efficient removal of damaged cells [4]. Apoptosis is regulated by many factors, receptors, genes and signaling pathway elements [5]. Abnormal apoptosis plays an important role in the pathogenesis of many diseases [6]. There is a loss of balance between proliferation and cell death in HCC, which represents a protumorigenic principle [5]. Notably, reduced apoptosis is associated not only with the progression of HCC, but also with tumor resistance to treatment [7]. In this study, based on mRNA profiling data collected from the cancer genome atlas (TCGA) database, this study aimed to establish a signature based on apoptosis-related genes (ARGs) to predict the prognosis of HCC patients.

## Materials and methods

### Datasets collection and data preprocessing

TCGA mRNA-seq data and corresponding clinical data of 368 HCC patients were accessed from the UCSC Xena (https://gdc.xenahubs.net) [8] and selected as the discovery cohort. Then FPKM values were transformed into transcripts per kilobase million (TPM) values. The GSE76427 dataset was obtained from the gene expression omnibus (GEO) database [9] and used as the validation cohort. When multiple probes mapped to the same gene, we used the median values to represent the expression of that gene. Samples with missing or 0 days of OS were excluded. Meanwhile, the 160 ARGs list in “HALLMARK_APOPTOSIS” was downloaded from MSigDB.

### Unsupervised clustering and differentially expressed gene (DEG) analysis

Based on the similarity displayed by the expression levels of ARGs, the “ConsensusClusterPlus” package was used to classify patients with HCC into different subtypes (1,000 iterations and resample rate of 80%) [10]. Principle component analysis (PCA) was performed to evaluate gene expression patterns among different HCC subtypes. The Kaplan–Meier curve was used to illustrate the difference in survival among different HCC subtypes. The “limma” package [11] was applied to acquire the DEGs among different HCC subtypes with adj_*p* < 0.01 & |log_2_ (Fold Change)|> 1. David was employed to perform Gene Ontology (GO) classification and Kyoto Encyclopedia of Genes and Genomes (KEGG) pathway enrichment analysis (*p* < 0.05) [12].

### Build and validate of prognostic model of ARGs signature

In the discovery cohort, the univariate Cox regression analysis was used to screen for prognostic ARGs. Then, the LASSO Cox regression using “glmnet” package in R was applied to develop the ARGs prognostic signature for the HCC patients. The optimal value of the lambda penalty parameter was defined by performing 10 cross-validations. The risk score calculating formula is:

$$RiskScore=\sum_{i=1}^{n}{exp}_{i}*{\upbeta }_{i}$$

The exp_i_ means the expression levels of each ARGs, β_i_ is the corresponding regression coefficients [13]. The patients were divided into high-risk and low-risk groups based to median score. The Kaplan–Meier curve was used to compare the OS of high- and low-risk groups. In addition, validation is performed in the validation cohort [14]. Univariate and multivariate Cox regression analyses were conducted to determine whether risk score was an independent prognostic factor. Moreover, receiver operating characteristic (ROC) curve was performed using “timeROC” package in R to verify the accuracy of the prognosis [15]. Furthermore, the accuracy of risk score, cluster and alpha fetoprotein (AFP) in predicting patient prognosis at 1-, 3- and 5-year was analyzed in the discovery cohort.

### Tumor immune microenvironment (TIME) and gene set variation analysis (GSVA)

The ssGSEA algorithm was applied to quantify the relative infiltration levels of various immune cells in the TIME of HCC. The gene set for marking each TME infiltration immune cell type was obtained from the study of Charoentong [16]. The immunoscore and stromalscore for each patient were calculated with the ESTIMATE algorithm through the R “estimate” package. GSVA enrichment analysis using “GSVA” R packages was performed to investigate the difference on biological process between high and low risk groups. The gene set of “c2.cp.kegg.v7.2.-symbols” was downloaded from MSigDB database for running GSVA analysis. Differentially expressed pathways were identified using the “limma” package in R, and FDR < 0.01 & |log_2_ (Fold Change)|> 0.1 was considered as statistically significance.

### Construction of the nomogram

Nomograms were widely used for cancer prognosis. Based on age, gender, tumor (T), node (N), metastasis (M) and risk score, a nomogram was constructed using “RMS” package in R. Calibration curves was used to visually predict deviations between actual and predicted survival [17].

### Statistical analysis

All statistics were carried out using the R software (version 3.6.3). Wilcox test was used to screen for statistically differentially genes and immune cells [18]. In Kaplan–Meier curves, log-rank test was used to check the significant difference in OS between groups. A *p* value < 0.05 was set as statistically significant for all the analyses.

## Results

### Identification of two different subtypes in TCGA cohort

**Fig. 1:**
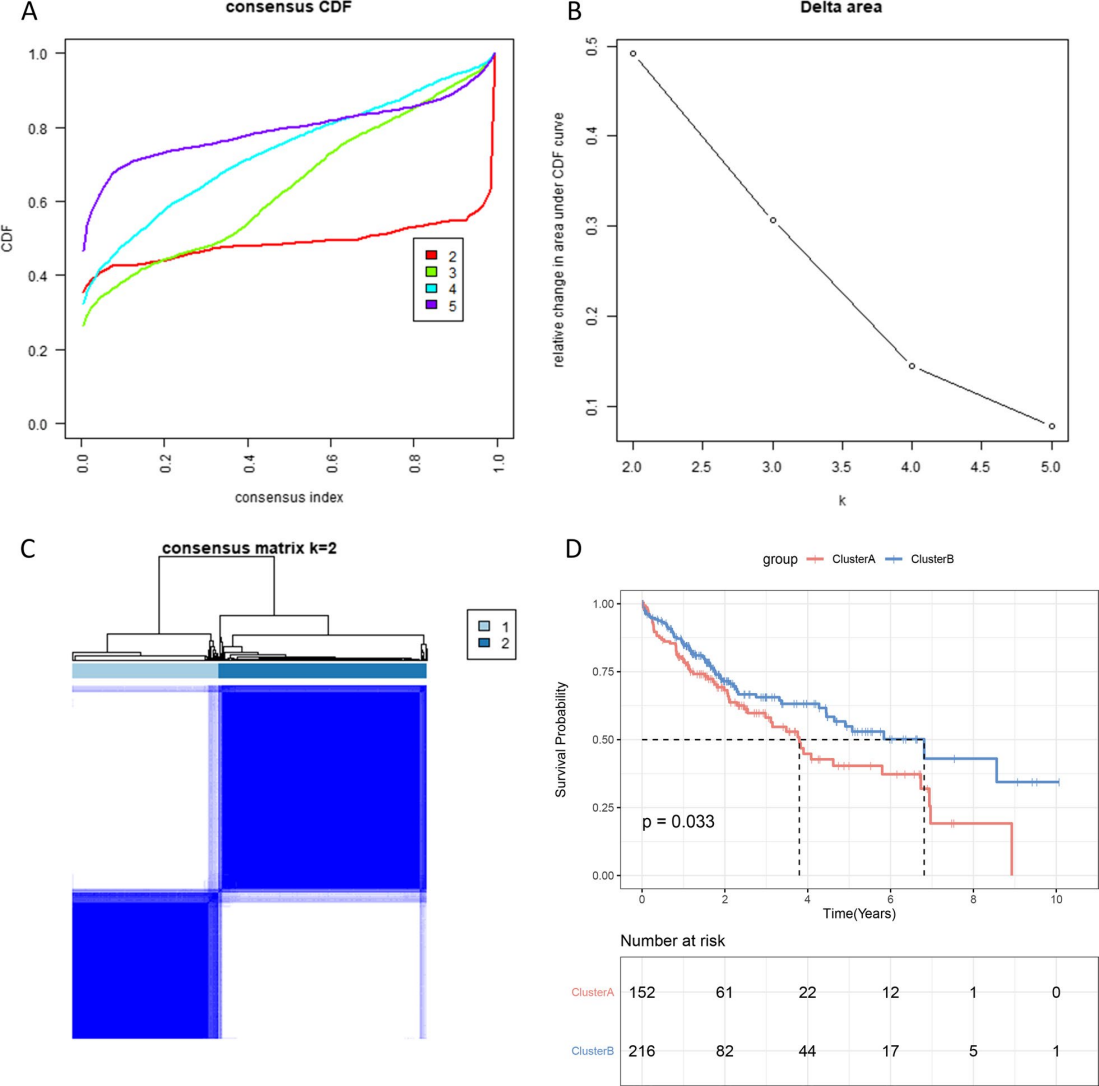
Consensus clusters by ARGs in TCGA cohort. **A** Consensus clustering cumulative distribution function (CDF) for k = 2–5. **B** Relative change in area under the CDF curve for k = 2–5. **C** Consensus clustering matrix for k = 2. (D) Kaplan–Meier curves of OS for patients with HCC in two clusters (cluster A/B)

**Fig. 2:**
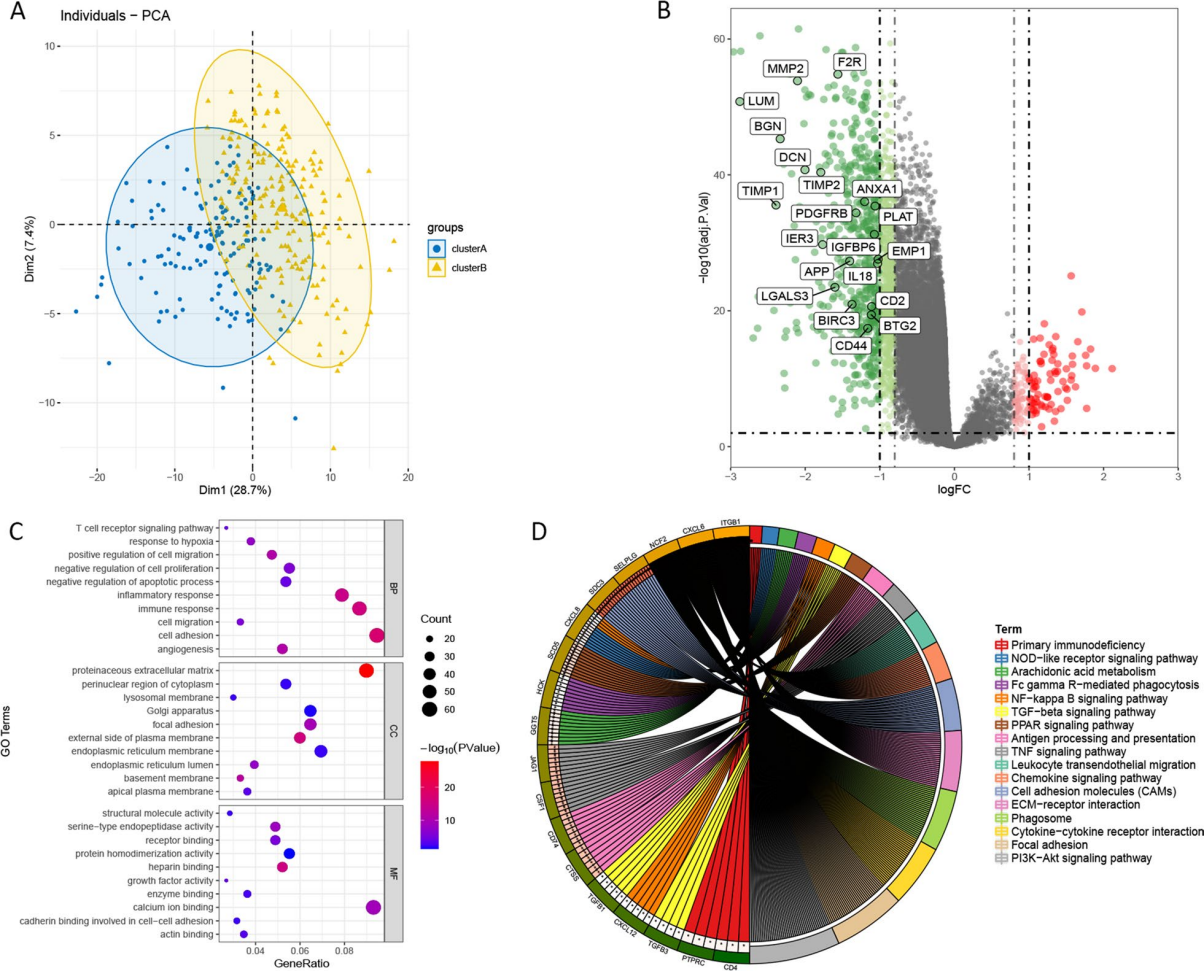
Differentially expression analysis between two different subtypes. **A** Principal component analysis of the total mRNA expression profile in patients with HCC. **B** The volcano plot of DEGs between cluster A and cluster B in HCC. **C** Significantly enriched GO terms of DEGs between cluster A and cluster B in HCC. **D** Significantly enriched KEGG pathways of DEGs between cluster A and cluster B in HCC

The k = 2 had higher intra-group correlation and lower inter-group correlation (Fig. 1A–B). Then, 368 patients with HCC were divided into two subtypes, cluster A (n = 152) and cluster B (n = 216) (Fig. 1C). Among them, cluster B has better OS (Fig. 1D). In addition, PCA analysis found that the gene expression profiles between cluster A and cluster B were well differentiated (Fig. 2A). A total of 643 DEGs between the two subtypes were identified (Fig. 2B). Among them, 569 DEGs were up-regulated in cluster A and 74 DEGs were up-regulated in cluster B. Functional annotation analysis indicated that these DEGs were mainly associated with biological processes such as cell adhesion, immune response, inflammatory response and cell proliferation (Fig. 2C–D).

### Construction of prognostic signature

**Fig. 3:**
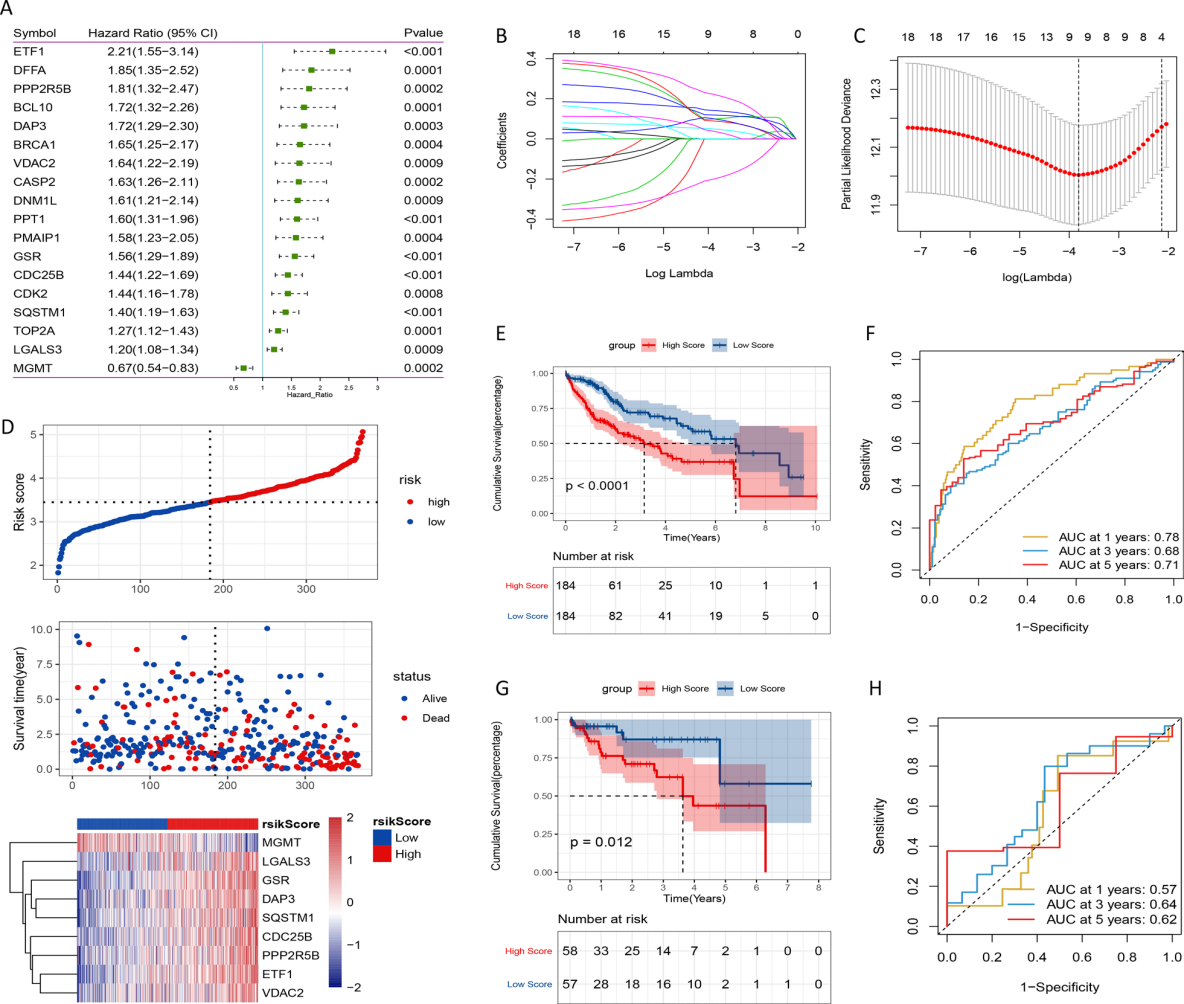
Construction of prognostic risk model in TCGA cohort. **A** Forest plot of ARGs associated with HCC survival. **B** LASSO coefficient profiles of the 18 ARGs in TCGA cohort. **C** Selection of the optimal parameter (lambda) in the LASSO model. **D** Distribution of risk score and OS status, and heatmap for nine model genes in the TCGA cohort. **E** Kaplan–Meier curves of OS for patients with HCC based on the risk score in the TCGA cohort. **F** ROC curves showing the predictive efficiency of the prognostic risk model on the 1-, 3-, and 5-years survival rate in the TCGA cohort. **G** Kaplan–Meier curves of OS for patients with HCC based on the risk score in the GEO cohort. **H** ROC curves showing the predictive efficiency of the prognostic risk model on the 1-, 3-, and 5-years survival rate in the GEO cohort

All 160 ARGs were subjected to univariate Cox regression analysis. Then, 18 ARGs significantly associated with the prognosis of HCC were identified (Fig. 3A). LASSO Cox regression analysis was performed for these significant ARGs (Fig. 3B). Subsequently, nine genes (CDC25B, DAP3, ETF1, GSR, LGALS3, MGMT, PPP2R5B, SQSTM1 and VDAC2) were screened to construct a prognostic model (Fig. 3C). Patients were divided into high- and low-risk groups based on the median risk score. The distributions of risk score of HCC patients and the relationships between risk score and survival time were visualized in Fig. 3D. Kaplan–Meier curves indicated that patients with low-risk score had significantly longer survival (Fig. 3E). The ROC curve analysis indicated that the 1-, 3- and 5-year AUC values were 0.78, 0.68 and 0.71, respectively (Fig. 3F). Consistent with TCGA analysis, the validation in GEO cohort displayed the similar results (Fig. 3G–H). Furthermore, we also perform validation on the international cancer genome consortium (ICGC) database. The results also showed that the survival of patients with low-risk score was significantly better than that of patients with high-risk score (Additional file 1: Figure S1A). The accuracy of risk score, cluster and AFP in predicting patient prognosis at 1-, 3- and 5-year was analyzed in the discovery cohort. ROC curve analysis and decision curve analysis (DCA) showed that the prediction accuracy of risk score at 1-, 3- and 5-year was higher than cluster and AFP (Additional file 1: Figure S1B-E). These findings suggested that the risk score model had good performance.

### Estimation of TIME cell infiltration

**Fig. 4:**
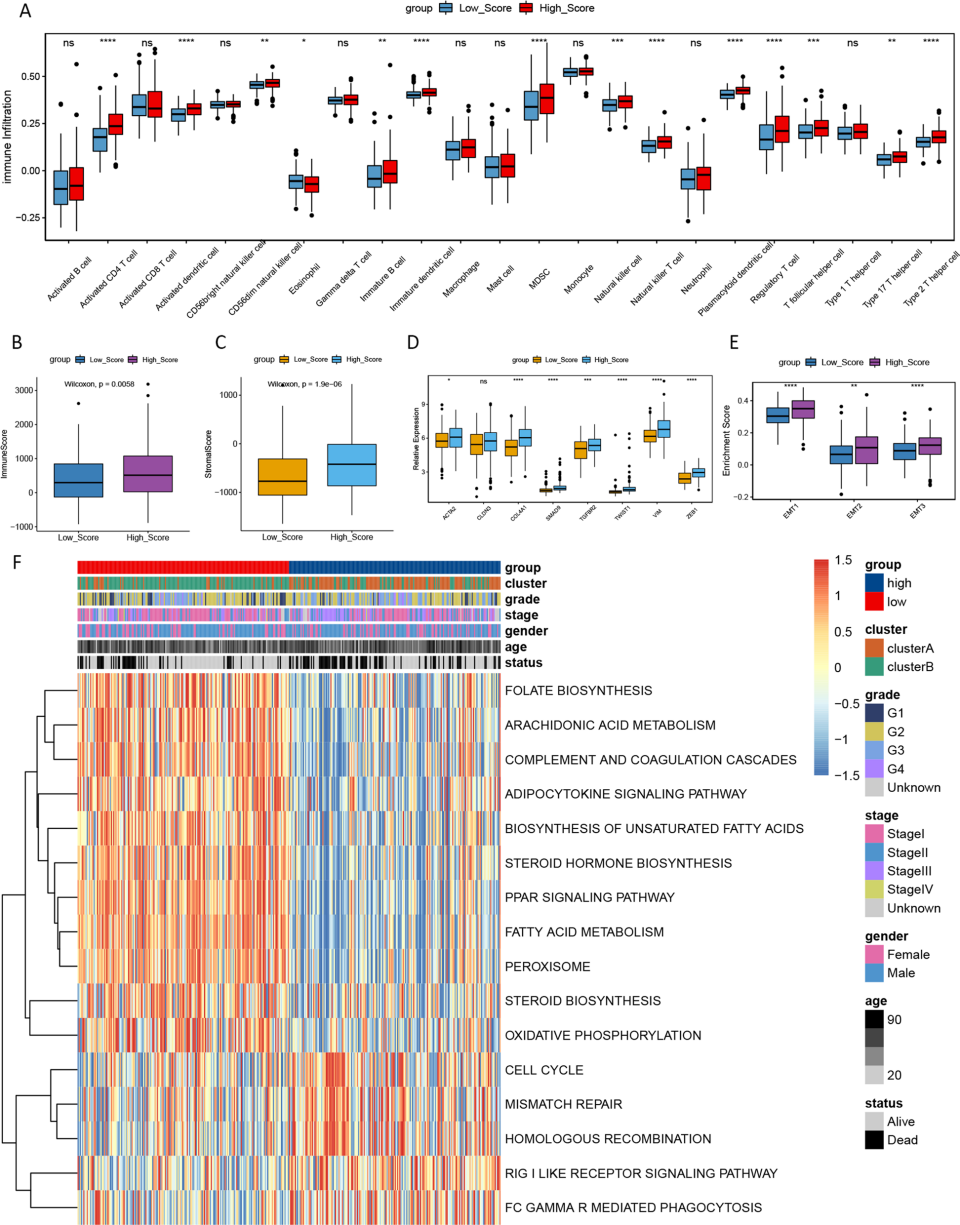
Characteristics of TIME cell infiltration between low and high risk group. **A** The abundance of each TME infiltrating cell in low and high risk group. The upper and lower ends of the boxes represented interquartile range of values. The lines in the boxes represented median value, and black dots showed outliers. The asterisks represented the statistical p value (*p < 0.05; **p < 0.01; ***p < 0.001). **B** Difference in immunescore between low and high risk group. **C** Difference in stromalscore between low and high risk group. **D** Difference in the TGF β-EMT pathway-related gene expression between low and high risk group. **E** Differences in stroma-activated pathways (EMT) between low and high risk group. **F** GSVA enrichment analysis showing the activation states of biological pathways in low and high risk group. The heatmap was used to visualize these biological processes, and yellow represented activated pathways and blue represented inhibited pathways

To explore the biological behavior between high- and low-risk groups, we conducted ssGSEA analysis. The results indicated that high-risk group was remarkably rich in immune cell infiltration including activated CD4 T cell, activated dendritic cell and natural killer cell (Fig. 4A). The similar results were observed in ESTIMATE analysis, with significantly higher immunoscore and stromalscore in the high-risk group than in the low-risk group (Fig. 4B–C). However, patients in the high-risk group did not show a matched survival advantage (Fig. 3E). We hypothesized that stromal activation in high risk group suppressed the antitumor effect of immune cells.

Then, we investigated the expression of chemokine and cytokine in both two groups. TGRB1, SMAD9, TWIST1, CLDN3, TGFBR2, ACTA2, COL4A1, ZEB1 and VIM were considered to be associated with the transcripts of transforming growth factor (TGF)-β/epithelial mesenchymal transformation (EMT) pathway. The results indicated that these cytokine and chemokine were significantly up-regulated in high-risk group (Fig. 4D). Subsequent analyses implied that EMT pathway was significantly activated in high-risk group (Fig. 4E). GSVA was performed to explore the difference on biological process between high- and low-risk groups. As showed in Fig. 4F, high risk group was markedly enriched in pathways associated with cell proliferation, such as cell cycle, mismatch repair and homologous recombination. All these findings confirmed our speculation.

In addition, the expression of commonly immune checkpoints, including PD-L1, CTLA-4, IDO1, LAG3, HAVCR2, PD-1, PD-L2, CD80, CD86, TIGIT and TNFRSF9, was investigated in high- and low-risk groups. The results showed that the expression levels of these molecules in the high-risk group were significantly higher than the low-risk group (Additional file 2: Figure S2A). Pearson correlation analysis between risk score and immune cells was performed and implied that the risk score was positively correlated with most immune cells (Additional file 2: Figure S2B).

### The risk score was an independent prognostic factor

**Fig. 5:**
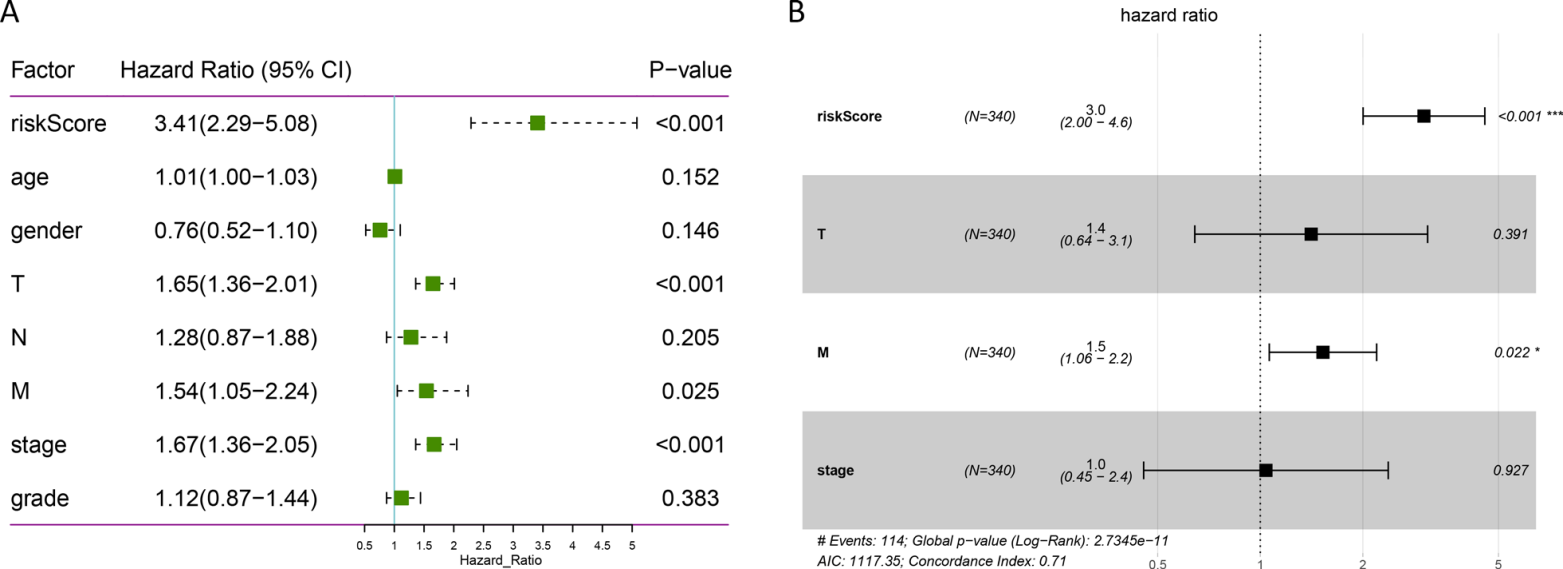
Univariate (**A**) and multivariate (**B**) Cox analyses

Univariate and multivariate Cox regression analyses were conducted to evaluate the prognostic value of the risk score. Univariate analysis indicated that T (*p* < 0.001), M (*p* = 0.025), stage (*p* < 0.001) and risk score (*p* < 0.001) were considerably associated with the OS (Fig. 5A). These factors were then included into multivariate Cox regression analysis, thereby showing that M (*p* = 0.022) and risk score (*p* < 0.001) remained closely correlated with the OS (Fig. 5B). In addition, univariate and multivariate analyses were performed in the ICGC database. The results also showed that the risk score was considerably associated with the OS (Additional file 3: Figure S3A and B). These findings demonstrated that the risk score generated from 9 ARGs was an independent prognostic factor for HCC patients.

### Construction of the nomogram

**Fig. 6:**
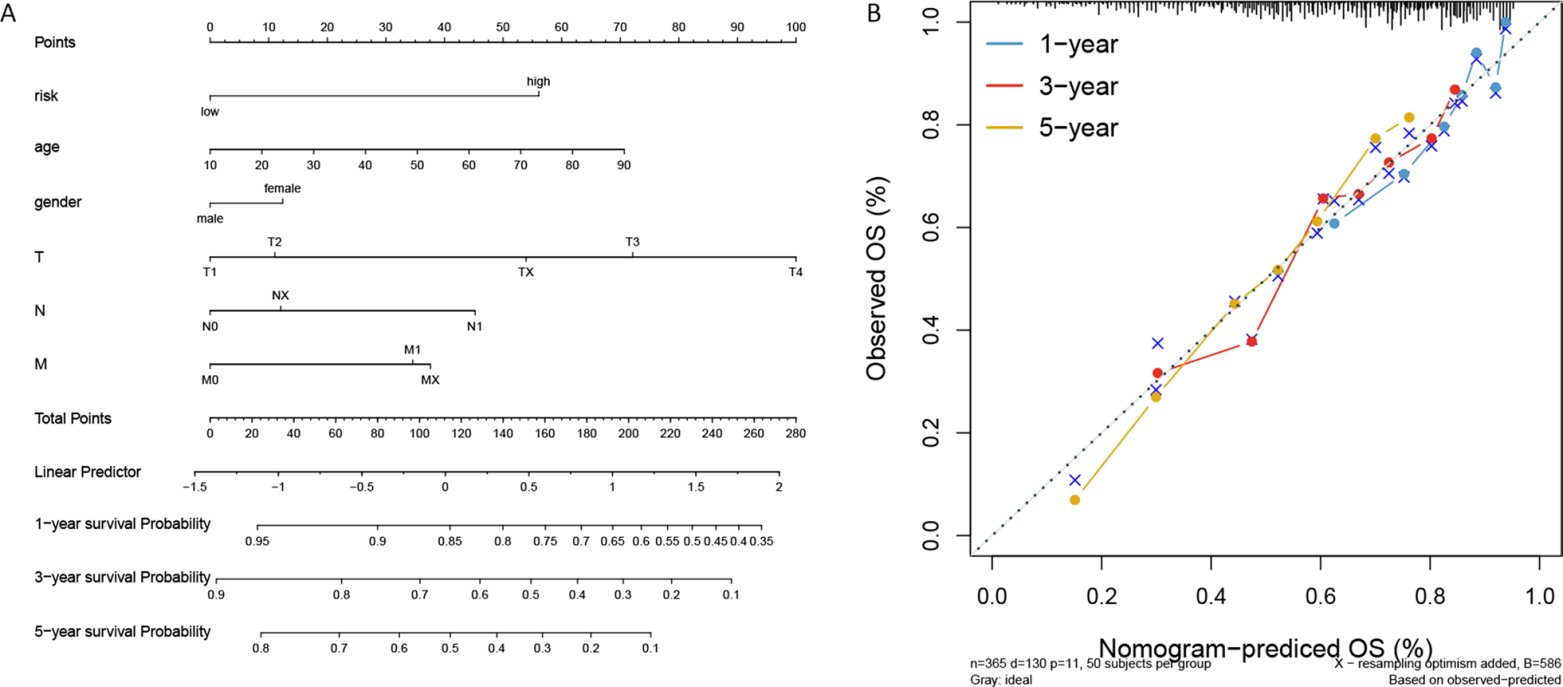
Nomogram (**A**) and calibration curve (**B**) in HCC

A nomogram was generated to predict the probability of 1-, 3- and 5-year OS, by incorporating age, gender, T, N, M and risk score (Fig. 6A). The 45° dotted lines represented an ideal model, and the calibration curves indicated that actual and predicted survival matched well (Fig. 6B). Subsequently, we also constructed nomogram based on ICGC database (Additional file 3: Figure S3C). Since the calibration curve of 5-years could not be obtained, we constructed the calibration curve for 1-, 2- and 3- year OS probabilities (Additional file 3: Figure S3D). Results showed that the nomogram-predicted probabilities (solid line) of 1-, 2-, and 3-year survival matched well with the idea reference line (dotted line).

## Discussion

Abnormal apoptosis of hepatocytes or accelerated proliferation activity of hepatocytes have been reported as important factors in the development of HCC or tumor progression [19]. Most studies have focused on the role of a single molecule in the diagnosis and prognosis of HCC [20–22]. In addition, some studies have established a risk model for predicting HCC prognosis based on the differential expression of ARG [23–25]. Although these risk models of ARG have the potential to predict the prognosis of HCC, few studies have linked them to the immune mediation of HCC. In recent years, the role of immunotherapy in the progression of HCC has become a research hotspot [26, 27]. In this study, using the expression data of HCC from TCGA database and the list of 161 ARGs from GSEA, a novel apoptosis-related signature prognostic-predictive model composed of nine genes (CDC25B, DAP3, ETF1, GSR, LGALS3, MGMT, PPP2R5B, SQSTM1 and VDAC2) was identified in HCC. The genes included in the signature were chosen by univariate Cox regression analysis and lasso Cox regression analysis. The Kaplan–Meier curve and ROC curve indicated that the risk score model based on nine ARGs had good prognostic performance. The prognostic power of risk score model was validated in GEO cohort. We also demonstrated that the risk score was an independent prognostic factor for HCC. A nomogram was constructed to help making individualized therapeutic strategy for patients as well. The characteristics of TIME cell infiltration, the expression of immune checkpoint expression, and the relationship between immune cells and risk score were also analyzed, which provided important ideas for further understanding the immunotherapy for HCC.

Cell division cycle 25B (CDC25B) is associated with mitosis and necessary for G2/M checkpoint initiation [28]. A case–control study in a HBV-related Chinese population indicated that CDC25B rs2295348 conferred a protective effect on HCC risk [28]. Mo et al. identified an mTORC1-associated gene signature containing six genes, including ETF1 and GSR, which can predict the prognosis of HCC [29]. Galectin-3, encoded by LGALS3, was identified to be a novel prognostic marker for HCC [30]. Several studies suggested that Galectin-3 was involved in metastasis-related processes in HCC [31, 32]. In addition, Zhang et al. identified a gene signature, including LGALS3, associated with the HCC microenvironment [33]. Liu et al. indicated a 5-gene signature, including PPP2R5B, based on ERS-related independent prognostic significance as a prognostic biomarker for HCC [34]. P62, encoded by SQSTM1, is an oncogenic protein aberrantly accumulated in HCC [35]. In mice, p62 is necessary and sufficient for HCC induction [36]. Saito et al. revealed that molecular targeting p62/SQSTM1 is a potential chemotherapy approach for HCC [37]. Li et al. established a six-gene signature, including SQSTM1, to predict OS in HCC [38].

The ssGSEA algorithm was applied to quantify the relative infiltration levels of various immune cells in the TIME of HCC. The results indicated that high-risk group was remarkably rich in immune cell infiltration. Significantly higher immunescore and stromalscore were observed in the high-risk group than in the low-risk group in the ESTIMATE analysis. However, patients in the high-risk group did not show a matched survival advantage. Tumors with an immune rejection phenotype also displayed a large number of immune cells, and these immune cells stay in the stroma surrounding tumor cell nests rather than penetrating the parenchyma [39]. The activation of stroma in tumor microenvironment is considered T-cell suppressive [40]. Moreover, we also found that the stroma activity was significantly increased in the high-risk group. Therefore, we hypothesized that stromal activation in high risk group suppressed the antitumor effect of immune cells. This suggests that ARG has a broad regulatory mechanism on the anti-tumor effect of immune cells. Cancer cells have the ability to activate different immune checkpoint pathways that harbor immunosuppressive functions [41]. Cancer immunotherapy targeting immune checkpoints is increasingly applied to multiple cancer treatment [42]. Co-inhibitory molecules (PD-1/PD-L1, etc.) can block the signal transduction process of T cells, thus inhibiting T-cell functions [42]. Recent years, PD-1/PD-L1 and CTLA-4 inhibitors have shown good therapeutic effects [41]. In our study, the expression of commonly immune checkpoints in the high-risk group was significantly higher than the low-risk group. These results may indicate that the immunosuppressive microenvironment in high-risk group may account for the poor prognosis.

In order to explore the differences in biological processes between high and low risk groups, GSVA enrichment analysis was performed using “GSVA” R packages. The results showed that the activities of pathways associated with cell proliferation, such as cell cycle, mismatch repair and homologous recombination, etc., were significantly increased in the high-risk group. During cell cycle progression, many regulators may be closely related to the early steps of carcinogenesis [43]. Radiation-resistant cancer cells can protect themselves by boosting their DNA-repair response [44, 45]. This further suggests that ARG has a broad regulatory mechanism on HCC progression.

Nomogram can be used to predict disease risk or prognosis by combining multiple indicators [14, 46]. A study showed that a nomogram combining clinical radiological risk factors and radiological features from hepatobiliary phase images could better predict the individualized risk of microvascular invasion of HCC patients [47]. Patients with high nomogram score were indicated for more aggressive treatment [48]. In this study, a nomogram that included risk score and other clinical features was constructed. The calibration curves showed good agreement between actual and predicted survival.

However, this experiment also has some limitations. First, the results of this experiment are obtained based on public datasets. Therefore, a large number of clinical samples need to be collected for validation. Second, the underlying molecular mechanisms revealed by this experiment need further study.

In conclusion, we constructed a prognostic signature comprising nine ARGs that could be used as a potential prognostic factor for HCC and help in clinical decision making for individualized treatment. The characteristics of TIME cell infiltration, the expression of immune checkpoint expression, and the relationship between immune cells and risk score were also analyzed, which provided important ideas for further understanding the immunotherapy for HCC.

## Supplementary Information

**Additional file 1**. **Figure S1**. Survival verification and comparison of prediction accuracy of risk score, cluster and AFP. (A) Comparison of survival between high and low risk groups in ICGC database. (B) The ROC curve analysis was used to analyze the 1-year prognosis prediction accuracy of risk score, cluster and AFP. (C) The ROC curve analysis was used to analyze the 3-year prognosis prediction accuracy of risk score, cluster and AFP. (D) The ROC curve analysis was used to analyze the 5-year prognosis prediction accuracy of risk score, cluster and AFP. (E) The DCA analysis was used to analyze 1-, 3- and 5-year prognosis prediction accuracy of risk score, cluster and AFP.

**Additional file 2**. **Figure S2**. Correlation analysis between immune-checkpoint related gene expression, immune cells and risk score. (A) Difference in the immune-checkpoint related gene expression between low and high risk group. (B) Pearson correlation analysis between risk score and immune cells.

**Additional file 3**. **Figure S3**. Univariate (A) and multivariate (B) analyses and nomograms (C) and calibration curves (D) in the ICGC database.

## Data Availability

All data generated or analyzed during this study are included in this published article. We searched for HCC public gene expression data and complete clinical annotations from GEO (http://www.ncbi.nlm.nih.gov/geo) and TCGA (https://tcga-data.nci.nih.gov/tcga/) databases. The accession numbers are GEO: GSE76427 and TCGA: HCC, respectively.
