# Supplementary figures and images for: Construction of a prognostic risk model based on apoptosis-related genes to assess tumor immune microenvironment and predict prognosis in hepatocellular carcinoma
Source: BMC Gastroenterol. 2022 Aug 26;22:400. doi: 10.1186/s12876-022-02481-w (PMC9414141; doi:10.1186/s12876-022-02481-w)

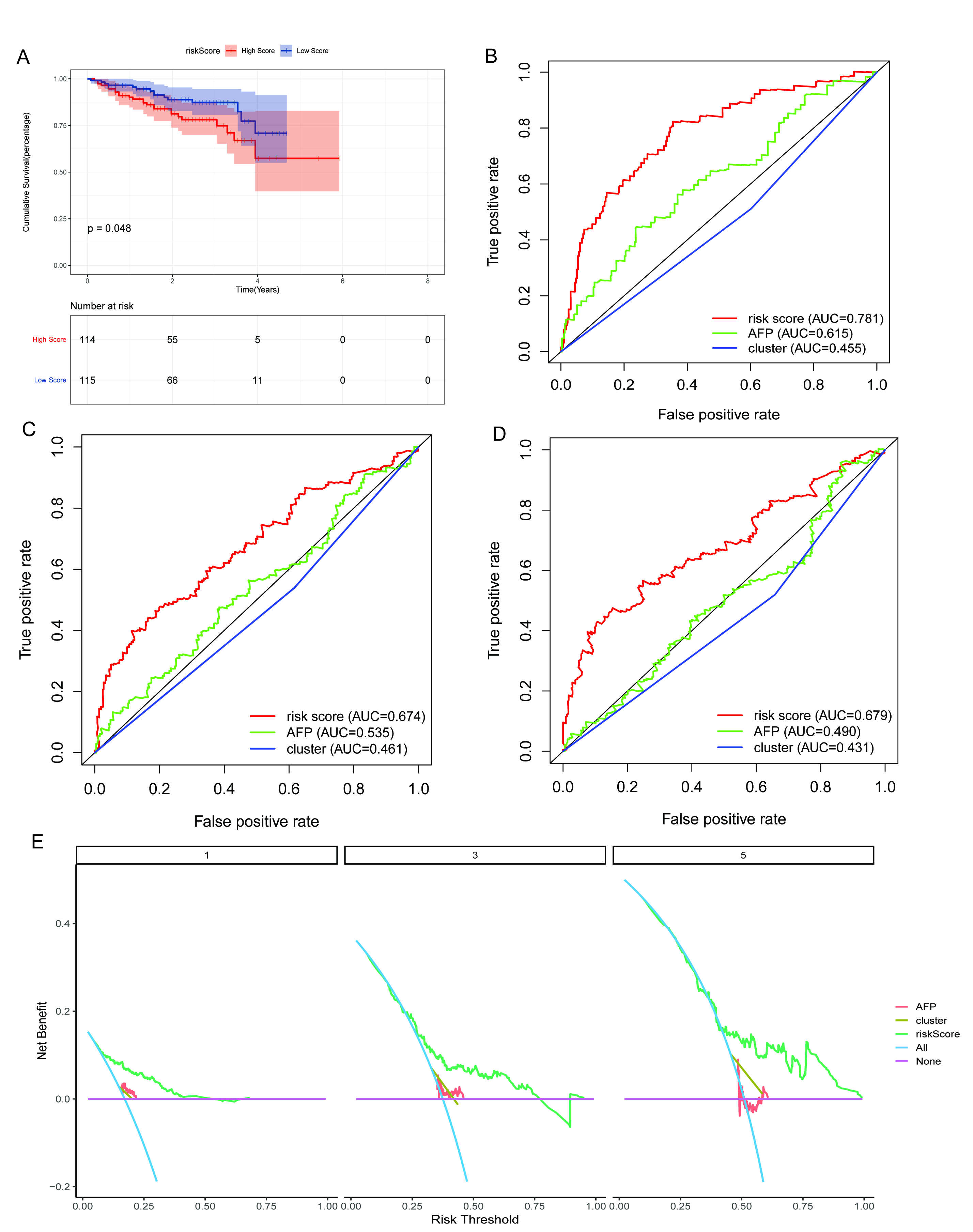

Supplement: Supplementary file 1 — Additional file 1. Figure S1. Survival verification and comparison of prediction accuracy of risk score, cluster and AFP. (A) Comparison of survival between high and low risk groups in ICGC database. (B) The ROC curve analysis was used to analyze the 1-year prognosis prediction accuracy of risk score, cluster and AFP. (C) The ROC curve analysis was used to analyze the 3-year prognosis prediction accuracy of risk score, cluster and AFP. (D) The ROC curve analysis was used to analyze the 5-year prognosis prediction accuracy of risk score, cluster and AFP. (E) The DCA analysis was used to analyze 1-, 3- and 5-year prognosis prediction accuracy of risk score, cluster and AFP. [file 12876_2022_2481_MOESM1_ESM.tif]

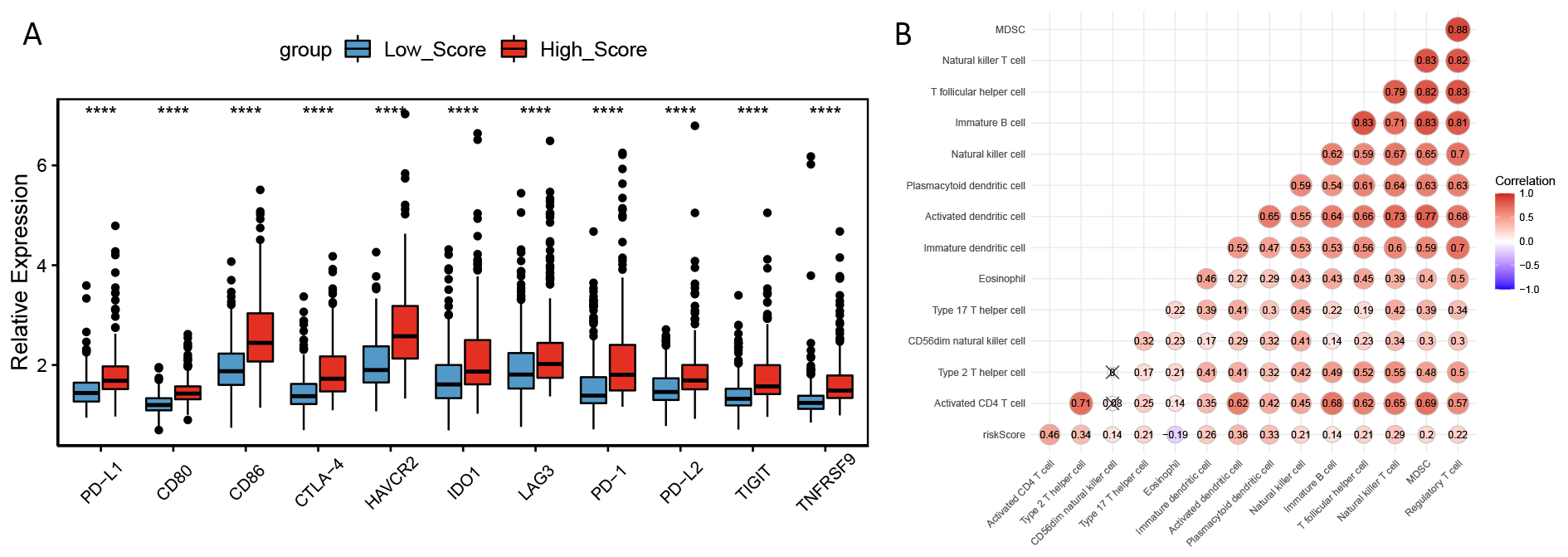

Supplement: Supplementary file 2 — Additional file 2. Figure S2. Correlation analysis between immune-checkpoint related gene expression, immune cells and risk score. (A) Difference in the immune-checkpoint related gene expression between low and high risk group. (B) Pearson correlation analysis between risk score and immune cells. [file 12876_2022_2481_MOESM2_ESM.tif]

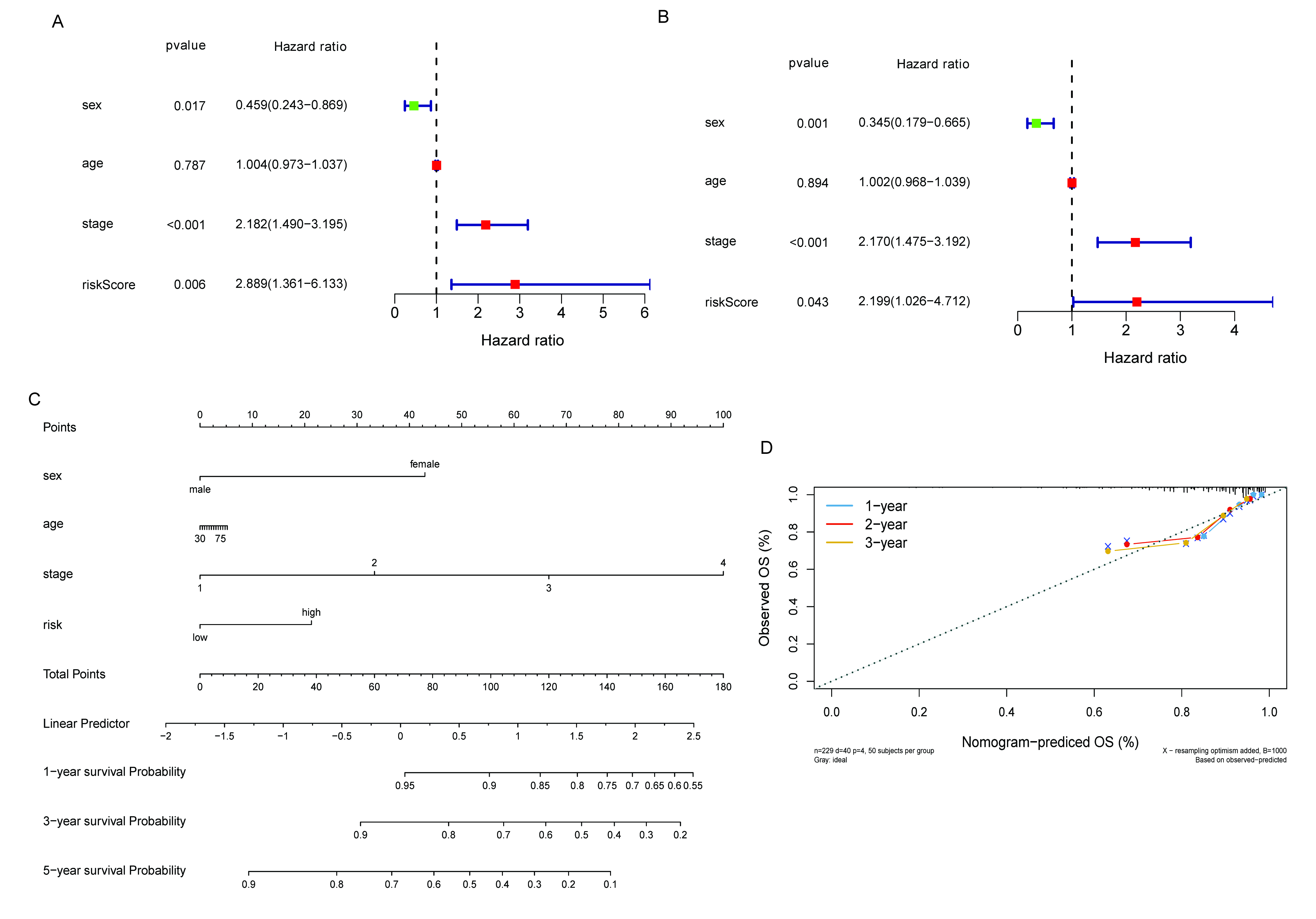

Supplement: Supplementary file 3 — Additional file 3. Figure S3. Univariate (A) and multivariate (B) analyses and nomograms (C) and calibration curves (D) in the ICGC database. [file 12876_2022_2481_MOESM3_ESM.tif]
